# Comparative analysis of plasma‐derived extracellular vesicles isolation methods in the context of neurodegenerative diseases

**DOI:** 10.1002/alz70856_098434

**Published:** 2025-12-24

**Authors:** Rafaela Luiza Costa Franco, Debora Afonso Silva Rocha, Thaís Lopes Pinheiro, Pedro Barbosa da Fonseca, Ananssa Silva, Victor Midlej, Fernanda G. Q. Barros‐Aragão, Luis E. Santos, Fernanda Guarino De Felice

**Affiliations:** ^1^ Federal University of Rio de Janeiro, Rio de Janeiro, RJ, Brazil; ^2^ D'Or Institute for Research and Education, Rio de Janeiro, RJ, Brazil; ^3^ Oswaldo Cruz Institute, Rio de Janeiro, RJ, Brazil; ^4^ Queen's University, Kingston, ON, Canada

## Abstract

**Background:**

Extracellular vesicles (EVs), particularly exosomes derived from plasma, are emerging as valuable tools in the search for non‐invasive biomarkers for early detection and monitoring of neurodegenerative diseases, such as Alzheimer's disease. However, isolating EVs from blood is challenging due to its complex protein content. Optimizing isolation methods is crucial to maximize yield and purity, improving biomarker detection and clinical translation. Thus, this study aims to compare EVs isolation techniques to determine the most efficient approach for detecting neurodegenerative biomarkers using the Simoa HD‐X platform.

**Method:**

Plasma samples were collected from 4 healthy volunteers aged 20–40 years. EVs were isolated using different methods: super centrifugation, precipitation (ExoQuick ULTRA), size‐exclusion chromatography (SEC ‐ Izon 70 nm) and SmartSEC, which combines SEC with an affinity‐based mechanism to capture protein impurities. To characterize the EVs and compare the isolation techniques, nanoparticle tracking analysis (NTA) was used to evaluate the concentration and size, while transmission electron microscopy (TEM) assessed morphology. The protein content of EVs was quantified using the Bradford assay. Immunoassays for EV markers and the ultrasensitive immunoassay Simoa HD‐X for neurodegenerative biomarkers in EV cargo, such as neurofilament light chain (NfL) and glial fibrillary acidic protein (GFAP), were performed.

**Result:**

EVs isolated from plasma showed distinct method‐dependent characteristics. NTA revealed predominant particle sizes around 100 nm for all approaches. SEC provided the highest particle yield, with a concentration of 10^14^ particles/mL in EV‐rich fractions. ExoQuick ULTRA and super centrifugation demonstrated high concentrations (10^13^ particles/mL), whereas SmartSEC showed lower concentrations (10^11^ particles/mL). TEM showed that SEC provided a high yield of particles and a greater size heterogeneity (100–1000 nm), while ExoQuick ULTRA was more pure by enriching specifically exosomes (30–150 nm). However, higher purity methods did not yield detectable biomarker levels when used with standard volumes of plasma, so additional biomarkers are being assessed.

**Conclusion:**

All methods are effective for isolating plasma‐derived EVs, but ExoQuick ULTRA and SEC demonstrated superior performance and are now being further evaluated for biomarker detection. These findings contribute to identifying efficient EV isolation methods, supporting advances in blood biomarkers for neurodegenerative diseases.

